# Electron Transfer Function versus Oxygen Delivery: A Comparative Study for Several Hexacoordinated Globins Across the Animal Kingdom

**DOI:** 10.1371/journal.pone.0020478

**Published:** 2011-06-01

**Authors:** Laurent Kiger, Lesley Tilleman, Eva Geuens, David Hoogewijs, Christophe Lechauve, Luc Moens, Sylvia Dewilde, Michael C. Marden

**Affiliations:** 1 INSERM U779, Universities Paris VI and XI, Le Kremlin-Bicêtre, France; 2 Department of Biomedical Sciences, University of Antwerp, Antwerp, Belgium; 3 Institute of Physiology and Zürich Center for Integrative Human Physiology (ZIHP), University of Zürich, Zürich, Switzerland; University of South Florida College of Medicine, United States of America

## Abstract

*Caenorhabditis elegans* globin GLB-26 (expressed from gene T22C1.2) has been studied in comparison with human neuroglobin (Ngb) and cytoglobin (Cygb) for its electron transfer properties. GLB-26 exhibits no reversible binding for O_2_ and a relatively low CO affinity compared to myoglobin-like globins. These differences arise from its mechanism of gaseous ligand binding since the heme iron of GLB-26 is strongly hexacoordinated in the absence of external ligands; the replacement of this internal ligand, probably the E7 distal histidine, is required before binding of CO or O_2_ as for Ngb and Cygb. Interestingly the ferrous bis-histidyl GLB-26 and Ngb, another strongly hexacoordinated globin, can transfer an electron to cytochrome c (Cyt-c) at a high bimolecular rate, comparable to those of inter-protein electron transfer in mitochondria. In addition, GLB-26 displays an unexpectedly rapid oxidation of the ferrous His-Fe-His complex without O_2_ actually binding to the iron atom, since the heme is oxidized by O_2_ faster than the time for distal histidine dissociation. These efficient mechanisms for electron transfer could indicate a family of hexacoordinated globin which are functionally different from that of pentacoordinated globins.

## Introduction

*Caenorhabditis elegans* is a free living, soil dwelling, nematode inhabiting the air/water interface of rotting organic matter where it feeds on bacteria and other micro-organisms [1]. Due to its small size (1 mm×80 µm) and the absence of a respiratory and circulatory system it is assumed that O_2_ reaches all tissues/cells by simple diffusion [2], [3]. In the soil, O_2_ tension can fluctuate over short distances and time spans from 21% to close to 0%, the latter e.g. coinciding with high bacterial (food) densities [4]. The metabolic rate of the worm is similar between O_2_ levels of 2–21%. Survival below 1% O_2_ needs the activation of hypoxia responsive pathways, whereas anoxia induces suspended animation. Hyperoxic conditions, normally lethal for most organisms within a short period, do not affect the worm's survival. Despite this broad O_2_ tolerance, *C. elegans* clearly prefers O_2_ tensions between 5–12%, avoiding higher or lower oxygen levels [5]–[9].

The maintenance of this O_2_ homeostasis requires O_2_ sensing and signalling pathways resulting in an adequate answer of the tissues/cells on variations in O_2_ tension. This is accomplished e. g. by the use of cGMP signalling through soluble guanylate cyclases (sGCs: αβ dimer) who are able to bind O_2_ through their PAS domain associated heme group (β subunit) and thus functioning as an O_2_ sensor. Different sets of sGCs are expressed by sensory neurons able to sense the variation in O_2_ concentration in the perienteric fluid. URX and BAG neurons detect respectively increasing and decreasing O_2_ concentrations and activate the sGCs gcy35/36 (URX) and gcy31/36 (BAG). The activation of these signalling pathways results in O_2_ depending social (aggregation) and feeding behaviour [10]–[16].

Globin-like molecules are other hemeproteins, in parallel to the sGCs/cGMP signalling pathways, that are involved in the sensing or metabolism of O_2_ and other small gaseous molecules (CO/NO). Extensive *in silico* screening of the *C. elegans* genome revealed at least 33 candidate globin molecules, all having orthologues in the closely related species *C. brigsiae* and *C. remanei*. These globin-like molecules show wide diversity in gene structure and amino acid sequence, suggesting a long evolutionary history. At the gene level, the number of introns (1–5) and their insertion positions (# 70) are very variable, in strong contrast with the conserved intron/exon pattern of vertebrate globin genes (2 introns; B12.2 and G7.0) [17]. At the protein level, their globin domains all display the invariant F8His whereas other determinants of the globin fold [18] are slightly relaxed. Additionally, GH interhelical and N- and C-terminal extensions frequently occur (see for instance a sequence alignment) [19] except for the globin sequence of GLB-1 (expressed from the *Caenorhabditis elegans* gene ZK637.13) which exhibits a similar size compared to that of a myoglobin (Mb). Nevertheless, significant sequence similarity with vertebrate α,β globin chains, Mb, Ngb and Cygb can be detected [19]–[24].

To date, 4 globins (GLB-1, GLB-26, GLB-6 and GLB-5 expressed respectively from gene C18C4.9 and C18C4.1) have been expressed in *E. coli* to characterize more precisely their functional properties. The crystallographic structure of two of them (GLB-1 and GLB-6) was solved [25]–[26]. GLB-1 is a high spin, pentacoordinated (HisF8-Fe) protein displaying an overall globin fold with an open heme pocket binding O_2_ reversibly. The structure is comparable with domain one of *Ascaris suum* perienteric fluid haemoglobin allowing reversible oxygen binding to the heme iron atom with a high affinity due to the stabilisation of the bound ligand by an additional hydrogen bond from B10Tyr. The protein is located in a small subset of neuronal cells and in the head muscular tissue and it is suggested that it regulates facilitated diffusion in these cells [25].

The globin domain of GLB-6 is unable to bind gaseous ligands to the hexacoordinated heme iron atom due to a reduced flexibility of the E-helix caused by e.g. the absence of a D-helix and an elongation of the E-helix shifting the distal residue to the E11 position [26]. GLB-6 is expressed in the RMG hub interneuron [15] and it is assumed that it serves a role as redox sensor that responds to oxidative stress, either directly or indirectly by the change in O_2_ concentration.

Foraging behaviour based on differences in O_2_ concentration is mediated by the neuropeptide receptor *npr-1* in combination with GLB-5. The latter is a hexacoordinated protein expressed in the sensory endings of URX, AQR and PQR where also NPR-1 and the subunits GCY-35 and GCY-36 of soluble guanylate cyclases, acting as oxygen sensors, are produced [7], [8], [10], [13]. The change in cGMP concentration results in the depolarisation of these neurons by cGMP-mediated gating of an ion channel that contains the TAX-4 subunit. The mechanism of regulation by GLB-5 is unknown.

*In vivo*, GLB-26 is predicted to be myristoylated at the N-terminus, is located in the stomato intestinal muscle and in the head mesoderm and is unchanged upon hypoxia but is upregulated upon anoxia [19], [20], [25]. Its function however is still not clear. Like GLB-6, recombinant GLB-26 is a low spin hexacoordinated (His-Fe-His) protein with an open heme pocket conformation unable to bind O_2_ reversible.

Previously the observation of a hexacoordinated globin was rare, but since the discovery of Ngb and Cygb, two human hexacoordinated globins, it has become more known. Despite the presence of hexacoordinated globins in different phyla, the function remains unclear. Ngb for example is expressed in neuronal tissues as well as in some endocrine tissues [27], whereas Cygb is expressed in many tissues, particulary in fibroblasts-like cells [27]. Several hypothesis for their function are raised, from O_2_ supply and consumption, ROS (oxygen reactive species) detoxication and the NO catabolism to specific enzymatic functions. If we consider their ability to react with O_2_, the most abundant *in vivo* diatomic ligand, both exhibit an O_2_ affinity comparable to that of Mb, but through a new mechanism involving a very high intrinsic affinity for O_2_ compensated by the competition with a distal E7 His for heme binding [28]–[31]. Rather than the standard ratio of on- and off-rates as for pentacoordinated globins such as Mb, the competition with the internal ligand results in an observed O_2_ affinity that depends on the ratio of the competing ligand affinities. This leads to an apparently typical globin with reversible ligand binding properties, but with different redox properties. Indeed for the same O_2_ affinity, the autoxidation rate is highly increased for a hexacoordinated form. For this reason the *in vivo* role of these globins is still being debated. A strong bond of the internal residue may indicate an electron transfer function, by analogy to the cytochromes, while a weak bond supports more a classical role of the binding of a diatomic ligand. Interestingly GLB-26 [25] and GLB-6 [26] are both strongly hexacoordinated as demonstrated by the low CO binding affinity which is considered to be a stable ligand for a globin.

In this work we present a kinetic study on the full length *C. elegans* GLB-26 ability to transfer an electron to Cyt-c and O_2_, in order to get a better understanding of these intriguing molecules. Interestingly enough, this globin shares functional similarities with *C. elegans* GLB-6, human Ngb and to a lesser extent with Cygb which are also strongly hexacoordinated with a distal histidine.

## Results

### Primary sequence analyses

As expected neither GLB-1 and GLB-26 nor other *C. elegans* globins contain a signal peptide for extracellular secretion. Rather an N-terminal myristoylation site was found at the second N-terminal amino acid Gly2 for GLB-26. Such a co-translational modification was also predicted for the putative globins of *C. elegans*: GLB-6, GLB-11, GLB-17 and GLB-20 with a high confidence level. Note that GLB-17 reveals an additional S-palmitoylation site found also in GLB-13, GLB-21 and GLB-29. This underlines that certain globins might be bound to the membrane lipids. Other globins might be cytoplasmic or nuclear and even mitochondrial since for instance a targeting sequence was predicted for GLB-18 and GLB-27. Ngb and Cygb were both predicted to be cytoplasmic as confirmed by numerous immunohistochemistry works. However a subcellular localization in the vicinity of the mitochondria in the retina cells has been proposed for Ngb [32] while for the Cygb its expression has been also observed in the nucleus [33].

### Tertiary and quaternary structures

**Figure 1:**
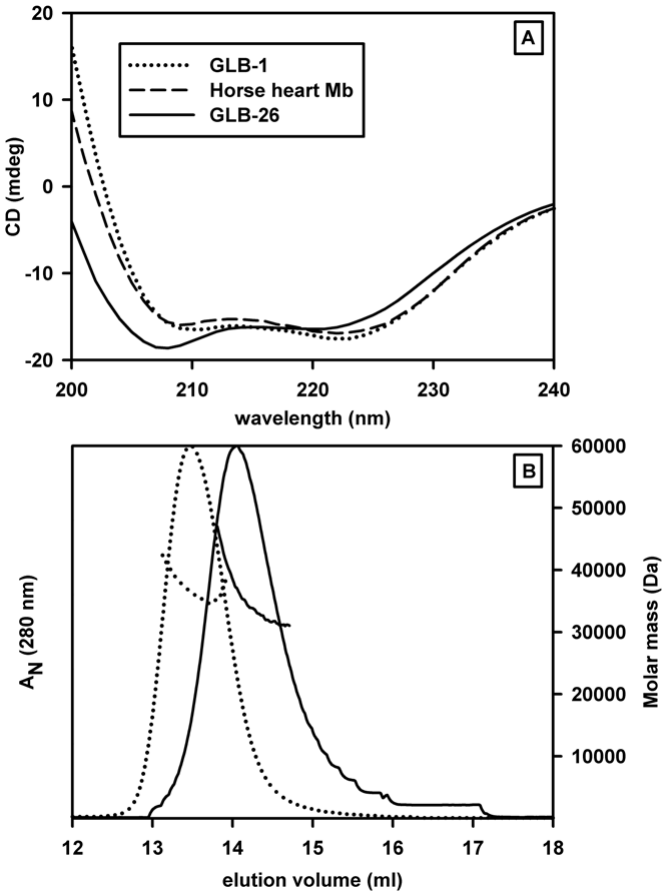
Tertiary and quaternary structures. A) Circular dichroism spectra for GLB-26 compared with those for GLB-1 and horse heart Mb. The deconvolution analysis using the CDNN deconvolution software gave about 85% of α helical form for GLB-1 and Mb and slightly less (70%) for GLB-26. B) Molar masses measured by multi-angle light-scattering after gel filtration on a Superose 12 HR 10/300 GL column for GLB-26 (dotted line) and GLB-1 (dashed line) proteins. Relative absorbance at 280 nm is given in solid lines. The elution profile of GLB-26 in its ferric form is displaced toward the lower molar mass species and the width of the elution profile peak is broader than that for fully dimeric oxygenated GLB-1.

To assess the proper folding of the recombinant proteins and in particular for a globin to evaluate the proportion of α-helix secondary structure, their circular dichroism spectra were recorded (Figure 1A). The full length GLB-26 displays an α-helical content of 70% slightly lower than for native horse heart Mb or GLB-1 (85%). This slightly lower α-helical content for GLB-26 can be explained by the presence of a less structured terminal sequences as previously observed for Cygb (N- and C- additional sequences) compared to Ngb [34] and other standard globin sequences such as Mb and GLB-1. In general, primary sequence analyses show that the extended regions and/or inter-helical regions contribute to a decrease of the overall α-helical fraction due to their coil form, although there are notable exceptions such as the *Bacillus subtilis* GCS globin domain [35], [36] and the recently solved structure of a protoglobin [37] which shows an additional Z-helix at the N-terminus.

The molar mass in solution of GLB-26 was determined by SEC-MALLS (Figure 1B). Under the conditions used, ferric GLB-26 occurs as an equilibrium of a monomeric (21 kD) and dimeric forms. Indeed, as compared to the fully dimeric GLB-1 [25], the elution profile of GLB-26 is displaced toward the lower molar mass species and the width of the elution profile is much broader. The presence of a weak interface could be of importance if the aggregation state of GLB-26 depends on the nature of the 6^th^ ligand (internal versus external) and eventually the ligation state (cooperativity behavior). Unfortunately the fast oxidation of the iron in the presence of O_2_ prevents a study of the quaternary structure of such ferrous ligation states. Note that the Ngb and Cgb studied in this work behave as monomers [38].

### Reduction of the heme iron atom

**Figure 2:**
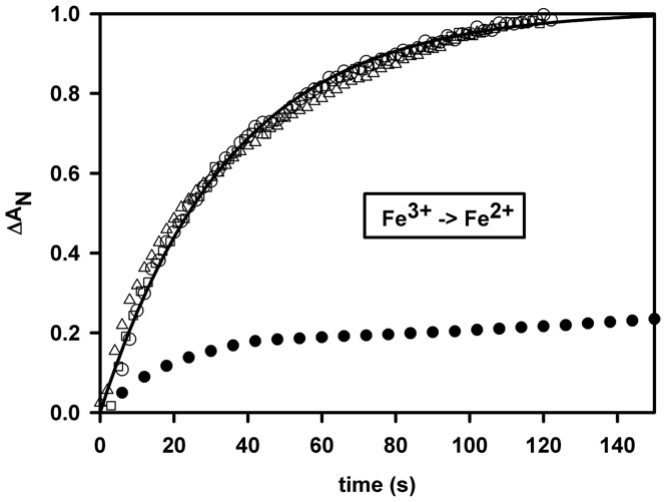
Anaerobic reduction. GLB-26 (white circles), human Ngb (white triangles) and Cyt-c (white squares) by 1 mM β-NADPH under continuous light exposure (30 W deuterium lamp of the HP8453 spectrophotometer). After turning off the light or under air exposure the globin return to its initial oxidized state (no transient oxy spectrum was observed). The reduction kinetics for GLB-26 and Ngb were quite similar with a rate equal to 0.028/s, as opposed to horse heart Mb (black circles) for which two thirds of the reaction was reached after 20 mn.

Several reducing systems such as dithiothreitol or sodium dithionite were tested to attempt to obtain the oxygenated form of GLB-26. These chemicals were added under anaerobic conditions just before sample equilibration under pure O_2_ on ice to prevent a fast reoxidation. Unfortunately it was not possible to generate a stable oxy form, even with the powerful system from Hayashi et al. [39] with ferredoxin as final electron donor. However, under anaerobic conditions the globin was easily reduced by sodium dithionite or by NADPH under illumination with a 30 W deuterium lamp (Figure 2); the latter reaction being as fast as for human Ngb or Cyt-c while for Mb the reaction was an order of magnitude slower.

### CO binding kinetics

The kinetics of CO rebinding were biphasic for GLB-26 and follow a simple model of competition between the distal histidine and CO for heme binding, with a slow phase that requires several seconds [25]. The full spectral variation of the slow phase was recorded with a diode-array spectrophotometer. This unambiguously validates and confirms the attribution of the slow transition to the replacement of the internal residue by CO based on the steady-state spectra. The [CO] dependence of the rebinding kinetics allowed us to extract a k_on_ value about 20000/s for the distal residue. Consequently the distal residue affinity for the heme is the highest known for a hexacoordinated species, an order of magnitude higher than the histidine affinity for human Ngb and two orders higher than that of human Cygb (Table 1). Indeed, for most of the hexacoordinated globins studied so far, the slow phase of replacement is quite small at 1 atm CO where usually k_on_CO>>k_on_His.

**Table 1:** Microscopic ligand binding rates for GLB-26 and electron transfer rates to Cyt-c and O_2_ compared to Ngb, Cygb, and Mb.

Ligand binding parameters & electron transfer rates	*C. elegans*GLB-26^§^	HumanNgb*	HumanCygb^⊥^	HorseMb^£^
k_on_CO (/µM/s)	20	50	0.65	0.5
k_off_CO (/s)	0.004	_	_	0.017
k_on_His (/s)	20000	1800	140	_
k_off_His (/s)	0.35	0.6	1.5	_
KHis	57000	3000	90	_
k_on_O_2_ (/µM/s)	40	170	2.5	14
k_off_O_2_ (/s)	_	0.5	0.9	11
KO_2_ (nM)	_	2.2	35	800
P_50_ O_2_ (Torr)°	_	6.8	1.8	0.4
e- transfer to Cyt-c(/µM/s) (this work)	10	15	0.02	0.003
e- transfer to O_2_ (/s) (this work)	2	0.025^$^	0.02^$^	0.00007^£^

Experimental conditions: 50 mM potassium phosphate buffer 0.1 mM EDTA pH 7.0, 25°C.*from [30] and for human Ngb after reduction of the intra-disulfide bond.**^§^**Data partially published from [25]. ^⊥^Data from [38] for human Cygb after reduction of the intra-disulfide bond. ^£^Measurement for Sperm Whale recombinant Mb in presence of 5 U/ml of catalase and SOD. °The O_2_ solubility coefficient was taken as 1.82 µM/torr. Note the distinction between the intrinsic affinity KO_2_ = k_off_/k_on_ (as for pentacoordinated forms) and the overall affinity for the ligand competition of the hexacoordinated globins obtained from the ratio of KO_2_ and KHis (k_off_/k_on_)/(1+K_His_)) and used here for the P_50_O_2_ calculation. ^$^these values are measured under air (35% O_2_ for GLB-26) and represents the lower limit of the autoxidation rate at 37°C; the rate increased at O_2_ tensions closer to the P_50_O_2_ for which the bimolecular oxidation of the hexacoordinated form by O_2_ prevails. A value of 0.8/s close to that of GLB-26 was measured for GLB-6 another hexacoodinated globin under air (21% O2) [26].

The slow thermal dissociation of CO was measured in the presence of pure O_2_ (Figure S1). Rather than a replacement reaction of CO by O_2_ an oxidation reaction was observed immediately upon CO release which points out that the oxygen binding is not stable.

### O_2_ binding kinetics

**Figure 3:**
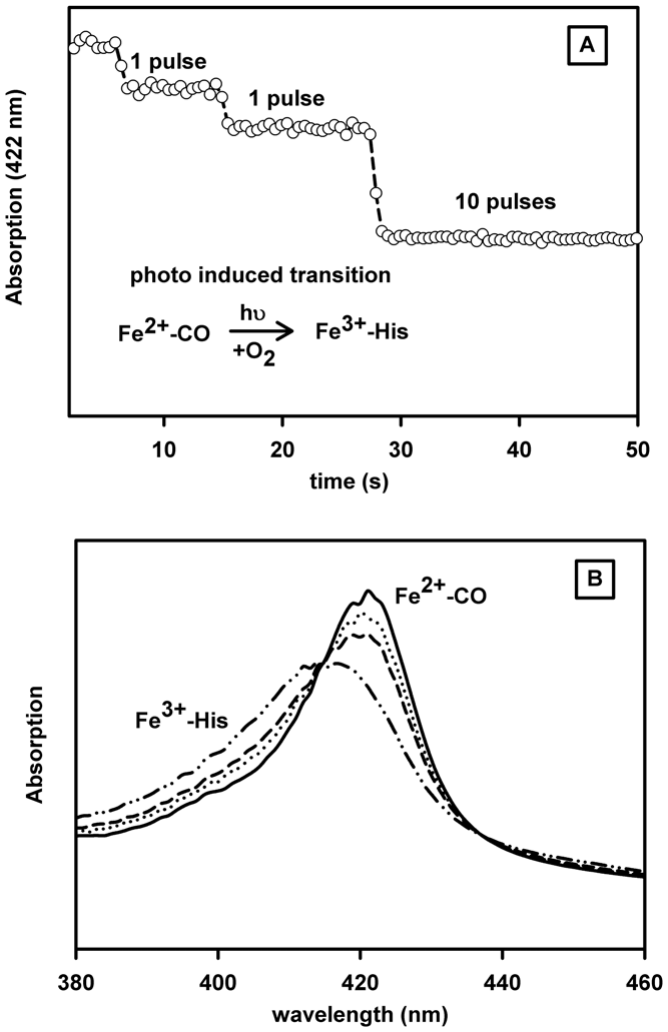
Oxidation by O_2_. Oxidation of GLB-26, induced by photodissociation of CO in the presence of oxygen (650 µM CO and 500 µM oxygen). A. After each laser pulse the fraction of heme which does not rebind first with CO undergoes a fast iron oxidation. B. The spectra recorded 0.5 s subsequent to the CO dissociation are typical of a transition from the steady-state CO to the oxidized spectra.

Flash photolysis allows a determination of the different microscopic binding constants for a hexacoordinated globin in which several competitive reactions occur for heme binding. For measuring the O_2_ binding in cases when direct photolysis of O_2_ is difficult, or as mentioned above when the oxy form is not stable, the use of a mixed O_2_/CO atmosphere allows a study of the association of O_2_ in competition with CO and eventually with an internal residue after flash photolysis. Note that the His binding parameters constants are first resolved in the presence of CO so that only the O_2_ parameters need to be determined. For systems where the oxygenated form is stable for at least several seconds, one obtains both the O_2_ association and dissociation rates. An attempt was made to measure the O_2_ dissociation rate for GLB-26 on samples equilibrated with O_2_/CO mixtures as described in the Methods section. Under these conditions a rapid oxidation of the GLB-26 iron is observed as evidenced by a decrease in signal after each O_2_ binding cycle initiated by photodissociation of the CO. The oxidation reaction after flash photolysis was unambiguously confirmed by monitoring the spectra 0.5 s subsequent to the photolysis with a diode-array spectrophotometer (Figures 3A & 3B). We can therefore discard the model of reversible O_2_ binding to GLB-26. Furthermore the high affinity for the distal His leads to a low affinity for the external ligand. So it is improbable that an oxygenated fraction at equilibrium can be present *in vivo* even in association with an efficient reducing system. Since the bis-histidyl hexacoordinated ferrous form is the reactive liganded state for an efficient electron transfer reaction, GLB-26 is likely involved in such redox reactions.

### Electron transfer to Cyt-c

**Figure 4:**
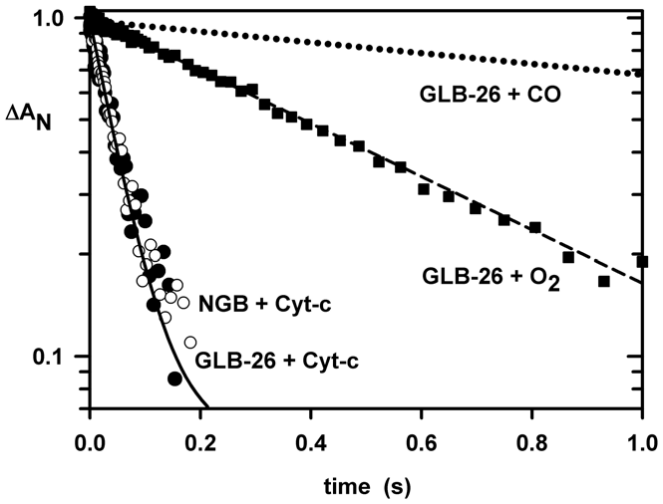
Electron transfer kinetics. Electron transfer from GLB-26 at 25°C. In parallel to the reversible CO and His binding (415 nm), additional shunting reactions were probed in the presence of Cyt-c (415 nm) or oxygen (410 nm). These reactions were initiated by photolysis of the CO form of 2 µM GLB-26. The pentacoordinated photoproduct has a lifetime of only 50 µs, so ms reactions shown here concern the His-Fe-His intermediate state. The top curve (dotted line) is for samples with only CO, which requires several seconds for the His to CO replacement reaction. In the presence of 2 µM Cyt-c, an electron transfer reaction occurs in a tenth of a second (black circles). A similar rate was observed for human Ngb (white circles). GLB-26 was equilibrated under CO (1 atm) and then diluted in a deoxygenated buffer containing Cyt-c (final [CO] was only a few µM); under this condition the His rebinding is much faster than that of CO allowing the formation of a bis-histidine hexacoordinated heme which transfers an electron to Cyt-c. In the presence of oxygen 1/3 atm and CO 2/3 atm (without Cyt-c) GLB-26 is oxidized in less time than the His dissociation, indicating an oxidation of the Fe-His complex without oxygen binding to the iron atom (black squares).

The electron transfer reaction between CO-GLB-26 and electron acceptors was initiated by CO photolysis from GLB-26. The pentacoordinated photoproduct has a lifetime of only 50 µs, so subsequent reactions concern the His-Fe-His intermediate state at low [CO]. Indeed, His rebinding is much faster than that of CO under these conditions, thus favoring the formation of the bis-histidyl hexacoordinated heme. Once the bis-histidyl species is formed the electron transfer reaction occurs rapidly at protein concentrations of a few µM. The bimolecular rate of transfer between GLB-26 and Cyt-c was 10^7^/M/s at 25°C. This electron transfer reaction was similar to that measured between human Ngb and Cyt-c (1.5×10^7^/M/s at 20°C; Figure 4), and that measured between deoxy Ngb and oxidized Cyt-c by stopped-flow under anaerobic conditions [40]. For both proteins the electron transfer does not require the dissociation of the distal His, as for the cytochrome family known to be tightly hexacoordinated. This reaction is more than four orders of magnitude faster than those for Cygb and deoxy Mb (Table 1) probably due to a weaker hexacoordination for Cygb (negligible for Mb) but also to a less favorable thermodynamic process since both exhibit a redox potential displaced toward the positive redox potential of Cyt-c [41], [42]. For Ngb and GLB-6, values of -129 and -192 mV have been reported respectively [26], [28]. Note that in cases where the bis-histidyl and the pentacoordinated forms may participate in electron transfer, CO binding always prevents the reaction.

The advantage of our methodology is a better time resolution; note that no electron transfer occurs as long as the CO is bound to the iron and generally the thermal CO dissociation is slow. This allows us to record several flash induced electron transfer reactions within a few minutes after a single sample mixing depending on the yield of CO dissociation from the heme toward the solvent after each flash (geminate versus bimolecular phases). In the presence of CO other reducing compounds can be used, instead of dithionite which can interfere with the oxidized Cyt-c, since we do not need to eliminate the remaining O_2_ traces as required for the anaerobic experiments.

### Oxidation of the bis-histidyl ferrous state

As described above, GLB-26 heme oxidation occurs quickly upon exposure to O_2_. After CO photodissociation, at a nearly equimolar O_2_/CO (concentration of about 500 µM), there is a first phase of competitive binding of the three ligands, namely the two diatomic ligands and the internal histidine, followed by a slow phase with rate of 2/s (Figure 4). This latter phase does not correspond to O_2_ replacement by CO, or that of replacement of the His ligand but to an irreversible oxidation of the Fe. The bimolecular rate for the reduction of the molecular O_2_ is 4 x 10^3^/M/s and is much slower than that for the electron transfer to Cyt-c.

**Figure 5:**
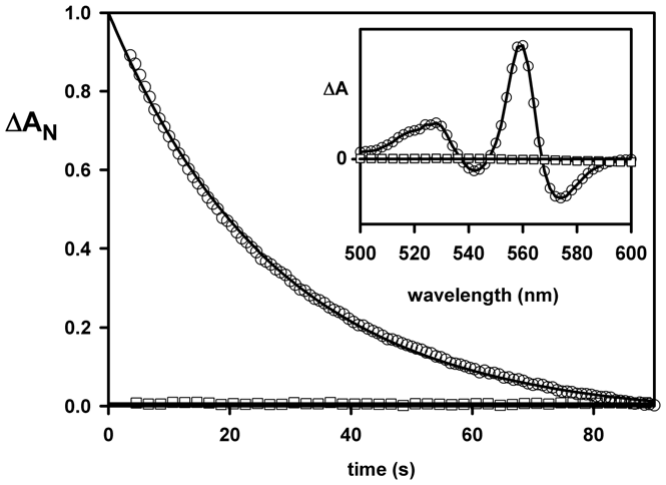
Reaction of ferrous His-Fe-His GLB-26 with CO or O_2_. GLB-26 was diluted in buffer saturated with CO: the slow absorption change in the visible region (560 nm minus 540 nm) is characteristic of the distal histidine replacement by CO (empty circles; the insert shows the variation of absorption between the spectra recorded few seconds after the mixing and after the completion of the replacement reaction). No such absorption change is observed by dilution into an oxygen saturated buffer (empty squares). Within a second after the mixing, faster than the time for His dissociation, deoxy GLB-26 is oxidized via electron transfer to the molecular O_2_. This confirms the fast ms electron transfer by an outer-sphere mechanism to O_2_ from ferrous His-Fe-His GLB-26, generated by CO photodissociation. Experimental conditions were 4 µM of globin in 25 mM potassium phosphate pH 7.0 at 8°C.

An alternate method to observe this reaction is to prepare a pure state of the ferrous hexacoordinated form by addition of dithionite, and then expose the sample to O_2_ or CO (Figure 5) at 8°C in order to obtain a full transition. Upon exposure to CO 1 atm, the replacement reaction of His by CO with rate approaching the His dissociation rate of 0.35/s at 25°C was observed. The same experiment with oxygen leads to rapid oxidation; note that this reaction occurs faster than the His dissociation (Figure 5), implying that the oxygen oxidizes the His-Fe-His complex by an outer-sphere mechanism, as opposed to the classical mechanism of a cycle of oxygen binding to the iron atom.

**Figure 6:**
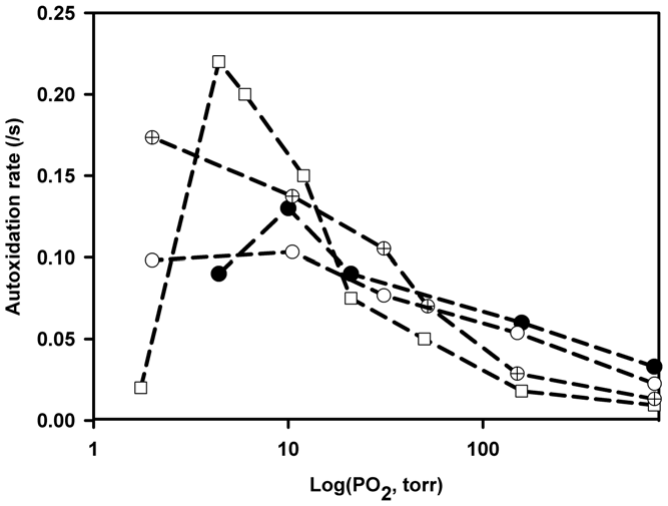
Rate of autoxidation versus PO_2_. Human Ngb WT (crossed circles), Ngb WT without disulfide bridge after reduction with DTT (white circles), human Ngb triple cysteine mutant Cys(CD7)→ Gly, Cys(D5)→ Ser and Cys(G19)→ Ser (black circles) and human Cygb double mutant Cys (B2)→ Ser and Cys (E9) → Ser (white squares). The acceleration of the rate of autoxidation for PO_2_ close to the P_50_O_2_ is assumed to be due to a bimolecular reaction between the ferrous hexacoordinated form and the molecular O_2_ while at high PO_2_ the dominant reaction is the unimolecular oxidation after O_2_ binding to the heme. Experimental conditions were: 25 mM potassium phosphate buffer, 0.1 mM EDTA, 37°C. Protein concentrations were between 2 and 4 µM.

A similar reaction might occur also for Ngb and Cygb and more generally for other strongly hexacoordinated globins under low oxygen pressures for which the bimolecular reduction is the dominant reaction (Figure 6). We observed at 37°C an increase of the autoxidation rate for Ngb (with or without the inter disulfide bridge) and for Cygb in the range of PO_2_ centered around their O_2_ affinity. By contrast at higher PO_2_ for which the deoxy hexacoordinated fraction is low the autoxidation rate decreases about ten times. The bimolecular mechanism of O_2_ reduction could proceed by an outer-sphere mechanism or after the histidine dissociation depending on the aging time of the hexacoordinated species. In fact it is technically difficult to distinguish between both mechanisms since the range of [O_2_] which can be investigated to assess the bimolecular regime for electron transfer between O_2_ and deoxy hexacoordinated globins is very narrow and also because a good absorbance signal impedes the use of submicromolar protein concentrations.

## Discussion

GLB-26 as well as Ngb, both strongly hexacoordinated globins, exhibit efficient electron transfer reactions with rates commonly attributed to cytochrome or oxidoreductase proteins. BLAST searches also show that GLB-26 shows homology with oxidoreductase domains. The experiments clearly indicate that the ferrous bis-histidyl hexacoordinated species is able to readily transfer an electron to Cyt-c or O_2_ while the ferrous His-Fe-CO form does not. The mechanism for electron transfer to O_2_ differs from what occurs with classical oxidase or oxygenase hemeproteins which are pentacoordinated. The iron hexacoordination in the globins with a distal histidine (allowing a nucleophile attack), e.g. GLB-26 and Ngb, could then be the reactive state for facilitating electron transfer. This is relevant to understand a possible role of these proteins with very slow internal ligand dissociation rates (several seconds).

It has been shown that the internal ligand rebinding in hexacoordinated hemoproteins occurs mainly at a consensus rate of a few picoseconds because of a barrierless formation of the heme-ligand complex while only a small fraction is allowed to migrate toward an “open” conformation accessible for an external ligand binding [43]. The O_2_ dissociation requires also seconds for Ngb and Cygb (Table 1). Shunting reactions which do not require dissociation of the ligand may thus provide a clue to the protein function. The reaction of NO with oxyHb is an example, where a rapid oxidation is observed in less time than the O_2_ dissociation [44]. The reaction of ferrous hexacoordinated GLB-26 and oxygen is a second example, displaying an oxidation without requiring dissociation of the His at least in its position compatible for ligand binding. Interestingly, the GLB-5 globin localized in the sensing neurons exhibits the spectral characteristics of a hexacoordinated heme in both ferric and ferrous states [13]. Similarly the O_2_ binding species is not clearly monitored by spectroscopy but one can see rather ferric to deoxy ferrous cycles of the globin suspension in presence of NADH, glucose, glucose oxidase and a reducing enzymatic system from *E coli*. In fact this result likely reflects that under air exposure GLB-5 is readily oxidized by O_2_ and that the ferrous hexacoordinated state appears only when the O_2_ is consumed by its reduction via the redox cycles of Fe^3+^/Fe^2+^ coupled with the reducing enzymatic system. The GLB-6 hexacoordinated globin shows no clear reversible binding of diatomic ligands. Exposure of the ferrous form to O_2_ leads to a very fast oxidation [26]. It is clear that for the hexacoordinated globins from *C. elegans*, the O_2_ binding is not necessary for electron transfer which can proceed by an outer-sphere mechanism. Indeed a similar bimolecular rate for the O_2_ reduction was measured for GLB-26 and GLB-6 (∼4×10^3^/M/s at 25°C) [26] and could approach the upper limit for this reaction rates since both have the highest affinity for the distal histidine so far reported.

This mechanism of fast bimolecular reduction of O_2_ could also be extended to other hexacoordinated globin such as Ngb and Cygb. The main difference for these latter globins is the reversible O_2_ binding which competes with the internal residue binding. This oxygen binding will somehow protect the bis-histidyl heme. At high [O_2_] the heme oxidation will proceed through a unimolecular reaction after O_2_ binding, while at low [O_2_] a bimolecular reaction will take place since the hexacoordinated form dominates. Indeed lowering [O_2_] from air saturation to the oxygen P_50_ increases the rate of oxidation for Ngb which is fully compatible with the bimolecular rate for O_2_ reduction observed for the *C. elegans* hexacoordinated globins. For the extreme case of GLB-26, O_2_ is readily reduced whatever the [O_2_], which can further participate in a biological redox reaction rather than H_2_O_2_ formation as a consequence of O_2_^-^ dismutation. Note that the formation of toxic ROS by further reaction of H_2_O_2_ with the heme is slowed by the strong hexacoordination of the ferric iron which then acts to inhibit the reaction. In the ferrous state this globin will consume O_2_ very efficiently as an oxidase, provided that a powerful enzymatic system is associated to ensure a redox cycle. Such a system has not yet been identified. Under anaerobic conditions it may be involved in an electron transfer reaction in view of the fact that it can reduce Cyt-c at a bimolecular rate corresponding to those between mitochondrial redox carriers. This redox reaction between two protein heme centers is also highly specific to Ngb and less to Cygb. Consequently a strong hexacoordination in a globin seems to induce oxido-reduction properties related to cytochromes, as opposed to pentacoordinated O_2_ carriers. This also allows efficient O_2_ reduction at low O_2_ concentrations, in contrast to a unimolecular process which is delayed by the His dissociation rate and generally by the competition with His for heme rebinding.

We must also consider globins which are partially hexacoordinated; that is, having a significant penta-coordinated population at equilibrium. A good example is provided by certain nerve globins from molluscs, annelids and marine nematodes [45], [46]. Nerve globins exhibit a Mb-like O_2_ affinity combined with a relatively low affinity for the distal histidine (0.1<K_His_<10) and a stable oxygenated form. Moreover their i*n vivo* concentration is high, in the range of one tenth to a few mM. These observations seem to confirm a function to facilitate the O_2_ flow under normoxic conditions and/or to act as an O_2_ reservoir under hypoxic conditions found in their habitat; the functional role of the reversible binding of the distal His is unclear. Based on this work we might propose a role in an electron transfer reaction since the redox potentials associated to the penta- and hexa-coordinated forms are likely different. One obvious redox reaction involves the iron atom and the O_2_ (linked to the O_2_ saturation level) which might be facilitated by a favorable redox potential as well as a preferential electron pathways mediated by the distal His (on the other hand, the reduction of the ferric heme might be facilitated after His release).

**Figure 7:**
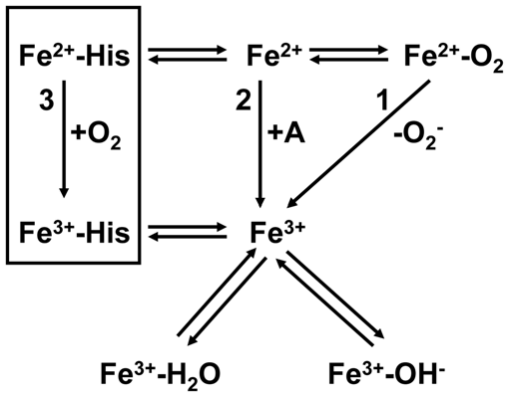
Main ferrous iron oxidation pathways for a globin. Pathway 1 is the superoxide dissociation (unimolecular process) which dominates at high O_2_ saturation level. Pathway 2 occurs at low O_2_ saturation level through a bimolecular reaction which does not require the formation of the Fe-O_2_ bond and can be mediated by a catalyst before transferring an electron to an acceptor molecule (“A”), different from the O_2_ one like Cyt-c another electron carrier. Note that the ferric iron is most often bound to a 6^th^ ligand: a water molecule, a hydroxyl anion or an amino-acid residue. Pathway 3 is different from 2 by the presence of a constitutive amino-acid tightly bound to the heme (generally a His at the E7 position). The electron transfer to O_2_ involves an outer-sphere mechanism since the His prevents its binding. Note that the ferrous bis-histidyl form of GLB-26 readily transfers an electron to Cyt-c so E7 His as a such may be also considered as a catalyst for electron transfer (pathway 2). The preferential pathway is modulated by the *in vivo* [O2] changes and its efficiency by the turnover rate of a coupling-regenerating system for reducing the ferric iron and to prevent further the formation of ferryl iron. Note that the oxidation of the Fe-O_2_ complex may also be triggered by bimolecular reaction with NO.

The main ferrous iron oxidation pathways for a globin are summarized in Figure 7. Pathway 1 is the superoxide dissociation (unimolecular process) which dominates at high O_2_ saturation level. Pathway 2 occurs at low O_2_ saturation level through a bimolecular reaction which does not require the formation of the Fe-O_2_ bond and is mediated by a catalyst. The electron acceptor could be also another electron carrier such as Cyt-c shown. The ferric iron is most often bound in equilibrium between a water molecule and a hydroxyl anion. Pathway 3 is only different from 2 by the presence of a constitutive amino-acid which prevents the oxygen binding.

For GLB-26, pathway 3 prevails over the other two. In the presence of O_2_, the Cyt-c reduction may occur after ligand dissociation in different ways: i) bimolecular electron exchange between proteins, or ii) bimolecular reaction with reduced molecular oxygen after globin autoxidation. Both reactions are presumed to coexist at low [O_2_] for which the globin is partially saturated and is susceptible to oxidation via bimolecular collisions with O_2_ rather than limited by the O_2_ dissociation at high [O_2_] [47]. The rate for the reaction of reduction of Cyt-c by GLB-26 after O_2_^-^ formation at 25°C pH 7.0 should be slower than that by direct transfer with the electron donor globin because the reduction of O_2_ takes a few tenths of a second. Note that the bimolecular rate for Cyt-c reduction with O_2_^-^ is equal to 10^6^/M/s as measured by the pulse-radiolysis technique [48]. Thus, the oxidation pathway is modulated by the *in vivo* [O_2_] changes and its efficiency by the turnover rate of a coupling-regenerating system for reducing the ferric iron and preventing further the formation of ferryl iron. Note that the oxidation of the Fe-O_2_ complex may also be triggered by bimolecular reaction with NO.

The same functional properties could be relevant for human Ngb and to a lesser extent Cygb. These proteins will probably be partially saturated at the low O_2_ tension found in the tissues and are then available for transferring one electron by collision with a partner molecule or via the molecular O_2_. The Ngb function due probably to its strong hexacoordination and its low cellular concentration is unlikely the Mb-like oxygen delivery, except possibly for the retina in which the concentration is higher than that of the other neuronal tissues. Indeed its retinal localization could be linked to the O_2_ consumption by the mitochondrial respiratory components of some distinct cell territories [32]. Inversely the localisation of Ngb in enriched mitochondrial tissues could also support an electron transfer function linked to the oxidative phosphorylation either with the electron carriers or with the oxidative reactive species produced by the organelle. Ngb might increase the neuron survival upon an oxidative stress by improving the mitochondria function [49] and reducing the oxidative stress effects. Furthermore there is currently no accurate cellular sublocalization of Ngb (or GLB-26) even though its cytosolic localization is clearly established. Proteins may be found partitioned between cytosol and mitochondria or the nucleus such as observed for Cygb [33]. Recently, it has been shown that neurons overexpressing Ngb exhibit a better protection against neurotoxic levels of H_2_O_2_ through a putative mechanism which could sustain the activation of the mitochondrial K_ATP_ channel and an increase in phosphoinositide-3 kinase mediated phosphorylation of protein kinase B [50]. The bis-histidyl hexacoordinated fraction *in vivo* for Cygb and Ngb will obviously depend on the variation of the tissue O_2_ tensions which can be displaced from the homeostatic ones upon an oxidative stress or hypoxia but also to their subcellular localization. The presence of an internal-disulfide bridge for both globins is also critical since the O_2_ affinity decreases by an order of magnitude upon its reduction [29], [38]. The O_2_ affinities are given in table 1. Their O_2_ affinity for the reduced form of the cysteines, that is, without the disulfide bonds is lower than that of Mb and in the range of O_2_ tensions commonly found in tissues between a few tenths to a few tens of Torr. Consequently the hexacoordinated fraction for Cygb and Ngb is likely high *in vivo* especially for the reduced state of the cysteines and may participate in other redox reactions. However the presence of the intra-disulfide bridge leads to a higher O_2_ affinity, similar to that of Mb [29], [38].

One cannot rule out a possible role for the hexacordinated globins in an oxygen sensing and signal transduction mechanism which can trigger a cascade of reactions to regulate the cell metabolism through the formation of a complex with a regulatory system. Note that certain hexacoordinated globins have extensions at the N and C-termini relative to Mb, such as Cygb or the different *C. elegans* globins except GLB-1, which could be involved in the protein localization and/or the interaction with another signaling or regulatory protein domain. Such a mechanism most likely requires a change of the ligation state to provoke a structural change and then favor binding to the partner protein or by contrast requires a change of the heme binding properties subsequent to the binding of a partner protein. On the other hand, electron transfer function could be faster, since the slow internal ligand dissociation is not required.

Since the autoxidation in the presence of O_2_ is much faster for the hexacoordinated vs pentacoordinated globins, especially in those studied from the *C. elegan*s genome, a possible involvement of its redox state in a regulatory mechanism as a redox sensor or in an electron transfer reaction may occur, as shown in this work. Since only small structural changes are expected from the hexacoordinated ferrous to ferric transition, the binding of a target molecule could change the internal ligand affinity in both or one of the redox states. An increased O_2_ affinity is not necessarily useful for O_2_ transport in contrast to an increase activity for the ROS catabolism due to a freely accessible heme. Indeed because of the competition with the internal His, the peroxidase activity is very weak for the hexacoordinated globins such as Ngb and Cygb [51]. An enhanced ROS detoxification activity for Ngb due the binding of a target molecule could explain why the expression of Ngb protects cells against an oxidative stress after an ischemia [52]. The fact that Cygb and the *C elegans* globins possess N and C-terminal extensions for possible lipid binding indicates a potential regulatory interaction. Such binding has been identified for the first time in the gills of a common littoral crab [53], which has an N-terminal extension with an N-myristoylation site as also found in GLB-26 and several other *C. elegans* globins. Similarly the crab globin is hexa-coordinated and even though its O_2_ affinity is compatible with that of an O_2_ carrier its fast autoxidation precludes this latter function.

In conclusion there are two functional classes of globin with regard to their ability to bind gaseous ligands reversibly or to participate in a redox reaction. A decade ago little was known about the hexacoordinated globins; pentacoordinated globins were the rule and essentially considered as O_2_ carriers even though redox reactions were also considered. One of the chemical reactions was the ubiquitous and efficient NO catabolism by dioxygenation with the globin oxygenated form. Today hexacoordinated globins appear to be widespread, from primitive invertebrates to mammals but also in plants and bacteria, and both globin types are found in the same species. Even though we cannot propose an unequivocal *in vivo* function for the hexacoordinated globins the present data allows us to discriminate between competing pathways. GLB-26 apparently plays a role in electron transfer, displaying efficient transfer to Cyt-c and molecular oxygen; this transfer can occur directly to the bis-histidyl hexacoordinated complex, rather than having to wait for dissociation of the internal ligand. GLB-26 and other hexacoordinated globins could play a role in the ROS chemistry or in the regulation of the mitochondrial activity or in other sensing functions as well.

## Materials and Methods

### Proteins

Human Ngb WT, human Ngb triple cysteine mutant Cys(CD7)→ Gly, Cys(D5)→ Ser, Cys(G19)→ Ser, human Cygb double mutant Cys (B2)→ Ser, Cys (E9) → Ser, GLB-26, GLB-1 and Sperm Whale Mb were expressed as recombinant proteins in *E. coli* and purified as described previously [28], [54]. Other proteins respectively Horse Heart Mb and Cyt-c were purchased from Sigma-Aldrich.

All experiments were performed in 50 mM potassium phosphate, 0.1 mM EDTA buffer at pH 7.0, 25°C.

### Sequence analyses

The PSORT II program [55] was used for searching possible subcellular localization, co- or post-translational modifications, for *C. elegans* globins. Prediction of membrane binding sites was performed with Myristoylator and CSS-Palm (www.expasy.org/tools/). PSIPRED program [56] was also used for prediction of the secondary structures from the globin sequence database.

### Spectra and circular dichroism

Spectra of the different liganded globin forms were recorded on a Varian Cary 400 or a HP8453 diode-array spectrophotometer. Circular dichroism (CD) spectra were measured with a JASCO J-810 spectropolarimeter equipped with a Peltier temperature regulation. Deconvolution analysis was done using the CDNN deconvolution software [57].

### Size Exclusion by Fast Protein Liquid Chromatography and Multi-Angle Laser Light Scattering (MALLS)

The protein molecular weight in solution was determined using on line multiangle laser light scattering coupled with size exclusion chromatography (SEC-MALLS). The gel filtration separation was carried out using Ettan™ LC liquid chromatography system (GE Healthcare) equipped with Superose™ 12 HR 10/300 GL column (GE Healthcare). Isocratic elution was performed at a flow rate of 0.4 ml/min using a mobile phase of 30 mM potassium phosphate buffer (pH 7.5), 100 mM NaCl, and 0.03% sodium azide at 25°C. Light scattering analysis was performed using an Ettan™ LC HPLC system with automatic degasser, thermostated auto-sampler, and connected in-line to a DAWN® HELEOS™ II 18-angle static light scattering detector (Wyatt Technology, Santa Barbara, CA) and to Optilab® rEX differential refractometer equipped with a Peltier temperature-regulated flow cell, maintained at 25°C (Wyatt Technology, Santa Barbara, CA). Calibration of the light scattering detector was subsequently verified using an albumin monomer standard (Sigma Aldrich). The molar mass for the protein was calculated from the light scattering data using a specific refractive index increment (dn/dc) value of 0.183 mL/g. The light scattering on the different detectors was analyzed using the ASTRA V software (Wyatt Technology, version 5.3.4.13) to finally obtain the absolute molecular weight and the hydrodynamic diameter for the eluted fractions.

### Reduction of the heme iron atom of GLB-26

Reduction of GLB-26 was attempted under anaerobic conditions by: (i) dithiothreitol (2 mM), sodium dithionite (0.1 mM) or the Hayashi's system (using ferredoxin as final electron donor) [39] (ii) by 1 mM β-NADPH under continuous light exposure (30 W deuterium lamp of the HP8453 spectrophotometer), before addition of pure O_2_ atmosphere at 5°C in order to prevent reoxidation. Progress of the reaction was measured after a few seconds of equilibration. No partial ferrous oxygenated spectrum was measured but only a transition from the deoxygenated toward the ferric hexacoordinated forms. Also note that GLB-26 reduced form is stable once saturated with CO.

### Ligand binding kinetics

Ligand binding kinetics for CO and c as well as the electron transfer kinetics between GLB-26 and Cyt-c were performed after laser photodissociation using a 10-ns YAG (Yttrium-Aluminum Garnet) laser pulse delivering 160 mJ at 532 nm (Quantel). The laser beam, as well as the monochromatic detection light, were transmitted to the sample optical cuvette through an optical cuvette by two optical fibers. For measuring the O_2_ binding to GLB-26 a mixed O_2_/CO atmosphere was used in order to study the association of O_2_ in competition with CO and eventually with an internal residue after flash photolysis. By mixing pure O_2_ and CO gases in near equal amounts, the sample is essentially bound to CO at equilibrium because the partition coefficient between both ligands is displaced toward the CO form as for most globins. After photolysis of CO, one expects to observe a rebinding phase between pentacoordinated hemes and the external and internal ligands in competition (the observed rate is the sum of the different rates for each ligand), followed by a slow phase of replacement of the bis-histidyl and oxygenated heme fractions by CO in order for the system to return to the CO bound steady-state. This phase depends on the bimolecular association rates for both external ligands and their respective dissociation rates. Generally, the CO dissociation occurs on a timescale slower compared to the O_2_ or the internal histidine dissociation and then can be treated as an irreversible process.

### Electron transfer studies

For the electron transfer studies a slight excess of dithionite was added to a concentrated globin preparation previously deoxygenated and further diluted into the final reaction mixture with the electron acceptor molecules either O_2_ or Cyt-c (in absence of O_2_). Because the final unreacted dithionite concentration is below 1 µM no bimolecular redox side-reaction occurs during our electron transfer kinetic timescales. The range of protein concentration was between 2 and 5 µM.

### Autoxidation

For measuring the autoxidation, dithiotreitol (4 mM) was used for reducing Ngb heme and disulfide bridge in the stock solution before monitoring the heme autoxidation after dilution to 1/100 into the phosphate buffer equilibrated at various PO_2_. The remaining DTT does not interfere with the oxidation reaction because the reduction of Ngb with DTT is much slower. Dithionite reduced GLB-26 (4 µM) was diluted in CO or O_2_ saturated buffer (25 mM potassium phosphate pH 7.0) at 8°C and the absorption change in the visible region (560 nm −540 nm) measured. Absorption difference immediately after mixing and after completion of the reaction was measured as well.

## Supporting Information

Figure S1 **CO dissociation from GLB-26.** CO dissociation after dilution of the carboxylated protein into an oxygenated buffer (1 atm O_2_). The rate limiting step of this reaction is k_off_ CO since the O_2_ binding occurs within a few µs and is immediately followed by the iron oxidation. The insert shows the variation of absorption during this reaction which corresponds to the difference of the steady-state spectra between CO and the oxidized hexacoordinated species.
(TIF)
