# Supplementary figures and images for: Electron Transfer Function versus Oxygen Delivery: A Comparative Study for Several Hexacoordinated Globins Across the Animal Kingdom
Source: PLoS One. 2011 Jun 1;6(6):e20478. doi: 10.1371/journal.pone.0020478 (PMC3106018; doi:10.1371/journal.pone.0020478)

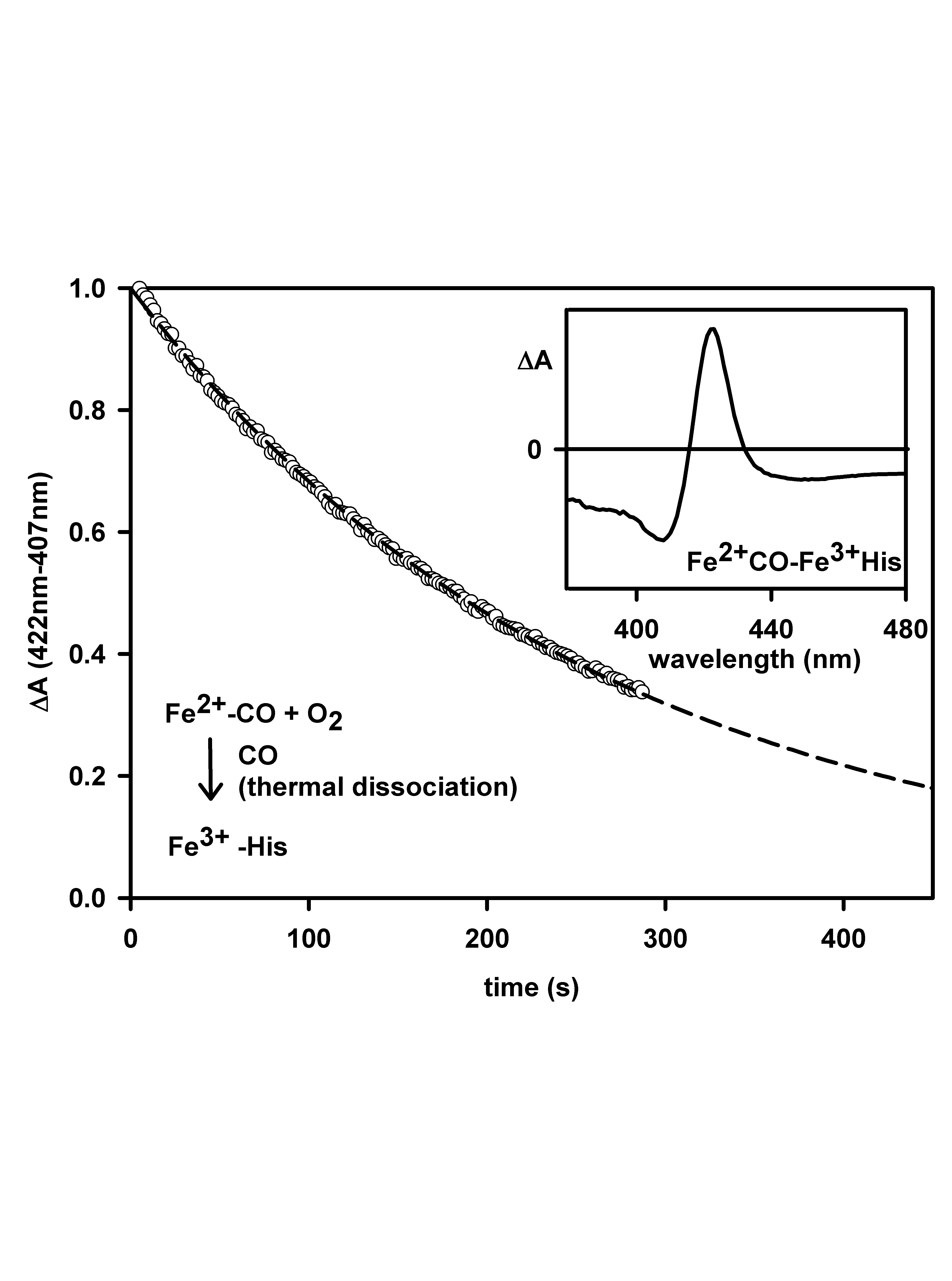

Supplement: Figure S1 — CO dissociation from GLB-26. CO dissociation after dilution of the carboxylated protein into an oxygenated buffer (1 atm O2). The rate limiting step of this reaction is koff CO since the O2 binding occurs within a few µs and is immediately followed by the iron oxidation. The insert shows the variation of absorption during this reaction which corresponds to the difference of the steady-state spectra between CO and the oxidized hexacoordinated species. (TIF) [file pone.0020478.s001.tif]
